# miR‐206 inhibits the growth of hepatocellular carcinoma cells via targeting CDK9

**DOI:** 10.1002/cam4.1188

**Published:** 2017-09-21

**Authors:** Chi Pang, Gang Huang, Kaili Luo, Yuying Dong, Fengtian He, Guankui Du, Man Xiao, Wangwei Cai

**Affiliations:** ^1^ Department of Biochemistry and Molecular Biology Hainan Medical College Haikou 570102 China; ^2^ Department of Biochemistry and Molecular Biology College of Basic Medical Sciences Third Military Medical University Chongqing 400038 China

**Keywords:** Apoptosis, cell cycle, target therapy, miR‐206, HCC, CDK9

## Abstract

miR‐206 plays an important role in regulating the growth of multiple cancer cells. Cyclin‐dependent kinase 9 (CDK9) stimulates the production of abundant prosurvival proteins, leading to impaired apoptosis of cancer cells. However, it is unknown whether CDK9 is involved in the miR‐206‐mediated growth suppression of hepatocellular carcinoma (HCC) cells. In this study, we found that the expression level of miR‐206 was significantly lower in HCC cell lines than that in normal hepatic cell line (L02). Meanwhile, CDK9 was upregulated in HCC cell lines. Moreover, miR‐206 downregulated CDK9 in HCC cells via directly binding to its mRNA 3′ UTR, which resulted in a decrease of RNA PolII Ser2 phosphorylation and Mcl‐1 level. Additionally, miR‐206 suppressed the cell proliferation, and induced cell cycle arrest and apoptosis. Similarly, silence or inhibition of CDK9 also repressed the cell proliferation, and induced cell cycle arrest and apoptosis. Taken together, the results demonstrated that miR‐206 inhibited the growth of HCC cells through targeting CDK9, suggesting that the miR‐206‐CDK9 pathway may be a novel target for the treatment of HCC.

## Introduction

Hepatocellular carcinoma (HCC) is a highly prevalent health risk and the third leading cause of cancer‐related deaths worldwide 1. HCC has become a serious threat to human health due to its rising incidence and high metastatic recurrence and mortality rates 2, 3. It has been shown that so many factors are involved in HCC development such as genomic and epigenetic alterations, abnormal gene expression, and signaling transduction dysfunction 4, 5, 6, 7. Generally, the mechanism of HCC development is still not clearly elucidated.

MicroRNAs (miRNAs) area class of endogenous 19‐22nt noncoding RNAs involved in post‐transcriptional regulation of gene expression via specific binding to the target mRNAs, leading to the translation inhibition or mRNA degradation 8. It is noted that miRNAs serve as tumor suppressor genes or oncogenes to control cell proliferation, invasion, migration, and apoptosis in multiple cancers including HCC 9. miR‐206, a member of miR‐1 family, is specifically expressed in skeletal muscle 10 and play an important role in skeletal muscle development, function, and pathology 10, 11. Furthermore, miR‐206 is involved in the pathogenesis of various diseases including pulmonary disease 12, heart failure 13, 14, Alzheimer's disease 15, 16, 17, and various types of cancers 18, 19, 20. It is frequently downregulated in some cancer tissues, and functions as a cell cycle regulator and tumor suppressor in clear‐cell renal cell carcinoma 18, gastric cancer 19, and rhabdomyosarcoma 20, indicating that miR‐206 plays an important role in the development and progression of cancer 21. Previous reports have shown that miR‐206 is significantly decreased in HCC tissues, and overexpression of miRNA‐206 can promote apoptosis, induce cell cycle arrest, and inhibit proliferation, invasion, and migration of HCC cells 22. However, the mechanism of miR206 in HCC development is not well defined.

Cyclin‐dependent kinase 9 (CDK9), the kinase of positive transcription elongation factor b (P‐TEFb), is crucial in the cell cycle control and regulation of apoptosis 23. CDK9 interacts with many transcription factors (TFs) and regulates the expression of antiapoptotic proteins for the survival of cancer cells 24. It is found that CDK9 is deregulated in most malignancies and is frequently upregulated in HCC 25. Inhibition of CDK9 activity can result in rapid downregulation of the short‐lived antiapoptotic proteins, inhibit cell proliferation, and induce apoptosis in HCC cells and other cancer cells 26, 27, 28, 29, 30. Therefore, targeting CDK9 may be beneficial to the treatment of HCC.

In this study, we found that miR‐206 significantly was decreased and CDK9 was markedly increased in HCC cells. Moreover, miR‐206 could downregulate CDK9 by directly targeting the 3′ UTR of its mRNA, leading to the proliferation suppression and apoptosis induction of HCC cells. These data indicated that miR‐206 plays its anti‐HCC effect via, at least partially, targeting the CDK9 signaling pathway, suggesting that miR206‐CDK9 pathway may be a novel potential target for the treatment of HCC.

## Materials and Methods

### Reagents

miR‐206 mimics and negative control (NC), miR‐206 inhibitor, and NC inhibitor were synthesized by RiboBio (Guangzhou, China). The sequences of the above RNA oligo were listed in Table S1. LDC000067 (CDK9 inhibitor) was purchased from Amquar Company (Shanghai, China). sh‐CDK9 plasmid was synthesized by GeneChem Company (Shanghai, China).

### Cell culture

Human HCC cell lines (HepG2, Bell7402, and HLE) and the human hepatocyte cell line (L02) were from American Type Culture Collection and cultured in DMEM basic medium (Gibco) with 10% fetal bovine serum (FBS, BI), 100 U/mL penicillin, and 100 *μ*g/mL streptomycin (Gibco) at 37°C in a humidified 5% CO_2_ incubator (Thermo Fisher Scientific).

### Plasmid construction

To construct the reporter and overexpression plasmids of CDK9, total RNA was extracted from HepG2 cells and reverse transcribed to cDNA by Thermo Fisher Scientific RevertAid TM First Strand cDNA Synthesis Kit (#K1622) with the primer oligo (dT)16. The 3′ UTR region of human CDK9 (1219 nt) containing miR‐206‐binding site was amplified by PCR using the cDNA as a template, and then inserted into the reporter vector pmir‐GLO (Promega), and the resulted recombinant plasmid was named as pmir‐CDK9. The mutant plasmid containing the 3′ UTR without the seed sequence of miR‐206‐binding site was constructed by using the site‐directed mutagenesis kit (Takara) and named as pmir‐CDK9‐M. A cDNA fragment for CDK9 open reading frame without 3′ UTR was amplified by PCR and cloned into the GV230 vector, and the resulted plasmid was named as GV230‐CDK9. The primer sets for plasmid construction were listed in Table S2.

### Transient transfection and establishment of stable cell lines

Bell7402 and HepG2 cells were seeded in six‐well plates and cultured for 18 h. Then the luciferase reporters (pmir‐GLO, pmir‐CDK9, or pmir‐CDK9‐M), or expression plasmids (GV230‐N1 or GV230‐CDK9) or RNA oligos (NC, miR‐206 mimics, NC inhibitor, or miR‐206 inhibitor) were transiently transfected into the cells with Lipofectamine 2000 reagent (Invitrogen) according to the manufacturer's instructions. To establish the stable cell lines overexpressing miRNA‐206 or sh‐CDK9 and the control cell lines, Bell7402 and HepG2 cell lines were cultured in the medium containing G418 after transfected with the plasmids GV230‐N1, GV230‐CDK9, GV251‐NC, GV251‐206, shCDK9‐NC, or shCDK9, respectively. The overexpression colonies of miRNA‐206 or sh‐CDK9 were selected in the presence of G418 for 2–4 weeks. The positive colonies were further identified by fluorescence microscopy analysis of GFP.

### Luciferase assay

HepG2 cell was seeded in 48‐well plates and grown to 80% confluence. Then the luciferase reporter (pmir‐GLO, pmir‐CDK9, or pmir‐CDK9‐M) (0.4 *μ*g/well) and miR‐206 or NC oligo (10 pmol/well) were transfected into the cells for 48 h. The cells were then lysed and the firefly and renilla luciferase activities were measured by Dual‐Luciferase Reporter System (Promega) according to the manufacturer's instructions. The firefly luciferase activity was normalized against the renilla luciferase activity. The transfection experiments were performed at least three times in triplicate. The data were represented as fold induction over the NC control.

### Examination of endogenous miR‐206 by quantitative PCR (qPCR)

Total RNA was extracted from cells with SanPrep Column microRNA Mini‐Preps Kit according to the manufacturer's instructions. Poly A polymerase was used to add poly‐A tails to the 3′ end of miRNAs (including miR‐206), and then the miRNAs tailed poly‐A were reverse transcribed by microRNA First Strand cDNA synthesis. Subsequently, the reverse‐transcribed miR‐206 was detected by the MicroRNAs Quantitation PCR Kit containing SYBR Green on the Stratagene M×3005P PCR Detection System (Agilent Technologies), using U6 small nuclear RNA as the internal reference. All reagents for the qPCR were purchased from Sangon corporation (Shanghai, China). The relative expression level of miR‐206 was determined with the 2^−∆∆CT^ method.

### Western blot

The HCC cells were seeded in six‐well plates and transfected with the plasmid (CDK9 over or empty vector CDK9‐NC, shCDK9 or empty vector shCDK9‐NC, 4.0 *μ*g/well) and/or RNA oligos (NC mimic, NC inhibitor, 206 mimic, or 206 mimic inhibitor, 100 pmol/well) for 48 h. Then, the cells were lysed by RIPA lysis buffer containing protease inhibitor cocktail (Roche), and the protein concentrations were determined by NanoDrop 2000 (Thermo Fisher Scientific). Subsequently, the total proteins (30 *μ*g/well) were separated with 10% SDS‐PAGE and transferred to PVDF (polyvinylidene fluoride) membranes (Millipore). After separately incubated with specific primary antibodies and horseradish peroxidase‐conjugated secondary antibodies, the signals on the membranes were visualized by enhanced chemiluminescence kit (Beyotime, China) according to the manufacturer's instructions. Tubulin was used as the loading control. Rabbit anti‐beta tubulin antibody (ab179513), rabbit anti‐MCL‐1 antibody (ab32087), rabbit anti‐RNA polymerase II CTD repeat YSPTSPS antibody (ab210527), rabbit anti‐RNA polymerase II CTD repeat YSPTSPS (phospho S2) (ab193468), rabbit anti‐CDK9 antibody (ab76320), and goat anti‐rabbit IgG H&L (ab6721) were from Abcam.

### Cell proliferation assay

A cell counting kit (CCK‐8) (Dojindo Laboratories, China) was used to analyze the cell proliferation. Briefly, the cells were seeded into 96‐well plates at a density of 2 × 10^3^ cells/well and cultured for 48 h. Then CCK‐8 reagent (10 *μ*L/well) was added to the cells and incubated for further 1 h at 37°C. Subsequently, the OD450 values were measured by Thermo Fisher Scientific Multiskan Spectrum. The optical density value of each well represented the proliferation of the HCC cells.

### Flow cytometry

The cell apoptosis was detected by PE Annexin V Apoptosis Detection Kit I according to the manufacturer's instructions (BD Pharmingen, China). All samples were analyzed using AccuriC6 flow cytometer (BD Accuri Cytometers, USA). Cell cycle phase distribution was determined in 70% alcohol‐fixed cells stained with 5 *μ*L PI‐RNase A staining buffer (Beyotime, China) for 30 min at room temperature, followed by analysis using AccuriC6 flow cytometer.

### Statistical analysis

All data were presented as the mean ± SD from duplicate or triplicate samples for each group. Comparisons between two groups were made by Student's *t*‐test, and comparisons among three groups were made with ANOVA, using the computer program GraphPad Prism (GraphPad Software Inc., USA). In all cases, a difference was considered statistically significant when the *P*‐value was <0.05.

## Results

### HCC cells express high level of CDK9 and low level of miR‐206

**Figure 1:**
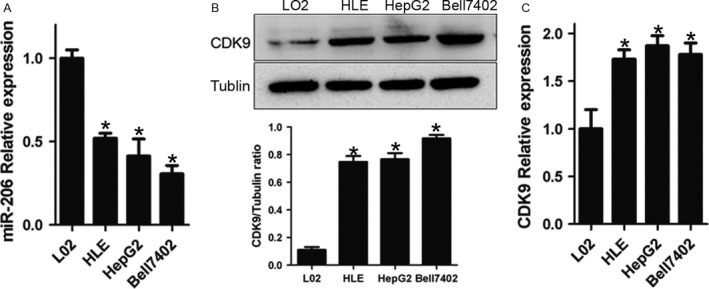
HCC cell lines express high level of CDK9 and low level of miR‐206. (A) qPCR analysis for miR‐206 expression level in above cell lines. Western blot and qPCR analysis for CDK9 protein (B) and mRNA (C) expression levels in L02, HLE, Bell7402, and HepG2 cell lines. The relative level of CDK9 expression determined with the 2^−∆∆CT^ method, and the data were represented as fold induction over the NC control. *P < 0.05 versus L02.

As shown in Figure 1, HCC cell lines expressed lower level of miR‐206 (HLE, *P *=* *0.0024; HepG2, *P *=* *0.0220; Bell7402, *P *=* *0.0029) and higher level of CDK9 mRNA (HLE, *P *=* *0.0101; HepG2, *P *=* *0.0083; Bell7402, *P *=* *0.0334) and protein (HLE, *P *=* *0.0047; HepG2, *P *=* *0.0049; Bell7402, *P *=* *0.0441) compared to L02 cells, indicating that there was an inverse relationship between the expression of CDK9 and miR‐206.

### 3′ UTR of CDK9 mRNA contains a functional target site for miR‐206

**Figure 2:**
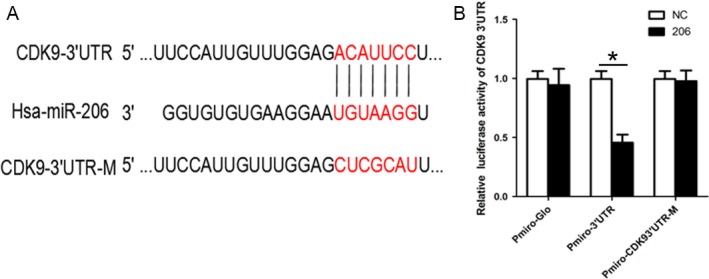
3′ UTR of CDK9 mRNA contains a functional target site for miR‐206. (A) A predicted binding site of miR‐206 in 3′ UTR of CDK9 mRNA was underlined. (B) HepG2 cells were cultured in 48‐well plates and were cotransfected with the reporter plasmids (pmir‐CDK9, pmir‐CDK9‐M, or empty pmir‐GLO) and RNA oligos (NC or miR‐206 mimics) for 24 h. Then the luciferase activity was determined with Dual‐Luciferase Reporter System. Firefly luciferase activity was normalized against the renilla luciferase activity. The transfection experiments were performed at least 3 times in triplicate, and the data were represented as fold induction over the NC control. *P < 0.05.

We employed three well‐used bioinformatics tools (TargetScan, miRanda, and PicTar) to predict the potential miRNAs which target CDK9, and miR‐206 was chosen finally. A potential miR‐206 target site (ACAUUCC) was within the 3′ UTR of CDK9 mRNA at 485‐491nt (Fig. 2A). To determine the biological function of miR‐206, luciferase reporter assays were performed in HepG2 cells with miR‐206 mimics (or NC oligo) and reporter plasmid (pmir‐GLO, pmir‐CDK9, or pmir‐CDK9‐M). As shown in Figure 2B, miR‐206 dramatically reduced the firefly luciferase activity of pmir‐CDK9 with the wild‐type 3′ UTR of CDK9 (*P *=* *0.0329), but had no effect on the luciferase activity of pmir‐CDK9‐M without the seed sequence of miR‐206‐binding site. Moreover, miR‐206 mimics had no effect on the firefly luciferase activity of empty pmir‐GLO. These results indicated that the target site of miR‐206 was within the 3′ UTR of CDK9 mRNA.

### miR‐206 downregulates the expression of CDK9

**Figure 3:**
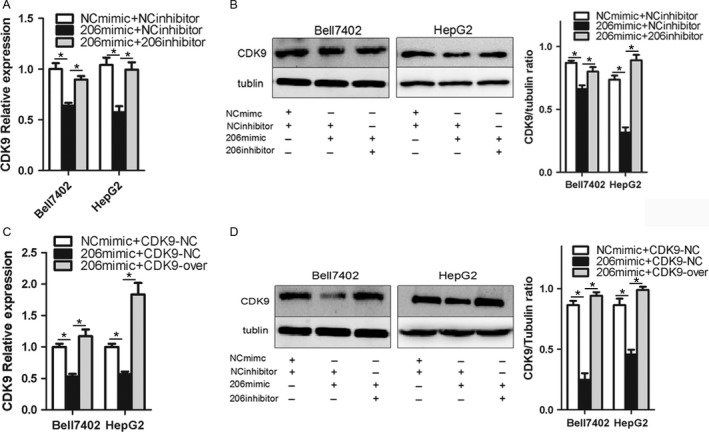
miR‐206 downregulates the expression of CDK9. Bell7402 and HepG2 cells were transfected with equal amount of indicated plasmids and/or RNA oligos for 24 h. Then the levels of CDK9 mRNA were assayed by qPCR (A, C) taking β‐actin as a control, and the protein level of CDK9 was detected by western blot (B, D) taking tubulin as the loading control. *P < 0.05.

As miR‐206 could bind to 3′ UTR of CDK9 mRNA, we checked the influence of miR‐206 on the expression of CDK9. As shown in Figure 3A and B, miR‐206 dramatically repressed the expression of CDK9 at mRNA (Bell7402, *P *=* *0.0014; HepG2 *P *=* *0.0222) and protein (Bell7402, *P *=* *0.011; HepG2, *P *=* *0.0156) levels in HCC cells, which were markedly attenuated by cotransfection of miR‐206 inhibitor (Bell7402, *P *=* *0.0393; HepG2, *P *=* *0.0026) (Fig. 3A and B) or GV230‐CDK9 without 3′ UTR of CDK9 (Bell7402, *P *=* *0.0415; HepG2, *P *=* *0.0072) (Fig. 3C and D). These results indicated that the suppressing effect of miR‐206 on CDK9 was specific.

### miR‐206 downregulates the expression of CDK9 and blocks the activation of RNA polymerase II

**Figure 4:**
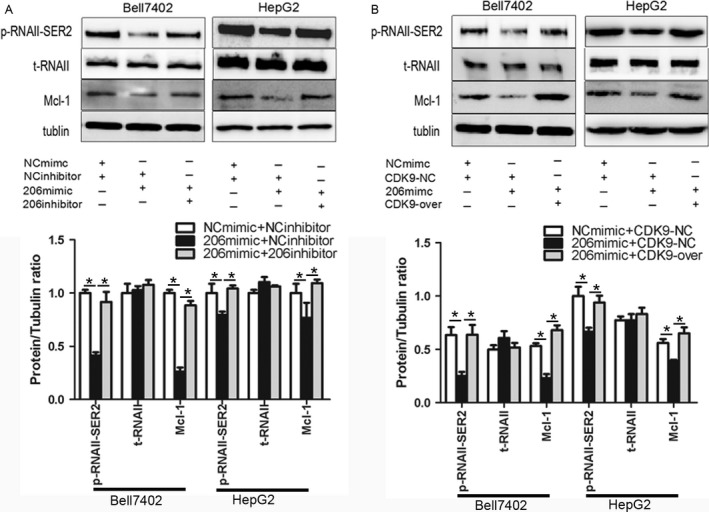
miR‐206 blocks the activation of RNA polymerase II. (A) Bell7402 and (B) HepG2 cells were transfected with indicated plasmids and/or RNA oligos for 48 h, then p‐RNAII, t‐RNAII, and MCL‐1 were measured by western blot taking tubulin as the loading control. p‐RNAII‐ser2, phospho‐RNA polymerase II ser2; t‐RNAII, total‐RNA polymerase II. *P < 0.05.

It is known that CDK9 is involved in regulating the phosphorylation of the carboxyl‐terminal domain (CTD) of the RNA polymerase II at Ser2, so we examined the phosphorylation changes of RNA polymerase II at Ser2 after treatment with miR‐206 in HCC cells. The results showed that transfection of miR‐206 markedly decreased the phosphorylation (Bell7402, *P *=* *0.0104; HepG2, *P *=* *0.0204), but not the total level of RNA polymerase II, which were rescued by cotransfection of miR‐206 inhibitor (Bell7402, *P *=* *0.0076; HepG2, *P *=* *0.043) (Fig. 4A) or GV230‐CDK9 (Bell7402, *P *=* *0.0001; HepG2, *P *=* *0.0314) (Fig. 4B). Furthermore, we investigated the influence of miR‐206 on the expression of Mcl‐1, the downstream gene of CDK9. As shown in Figure 4A and B, transfection with miR‐206 significantly suppressed the expression of Mcl‐1 in HCC cells (Bell7402, *P *=* *0.0026; HepG2, *P *=* *0.0197), which were also rescued by cotransfection of miR‐206 inhibitor (Bell7402, *P *=* *0.0103; HepG2, *P *=* *0.0125) (Fig. 4A) or GV230‐CDK9 (Bell7402, *P *=* *0.0272; HepG2, *P *=* *0.0098) (Fig. 4B). These results indicated that the miR‐206‐mediated downregulation of CDK9 reduced the activation of RNA polymerase II and inhibited the expression of Mcl‐1.

### miR‐206 inhibits the proliferation of HCC cells

**Figure 5:**
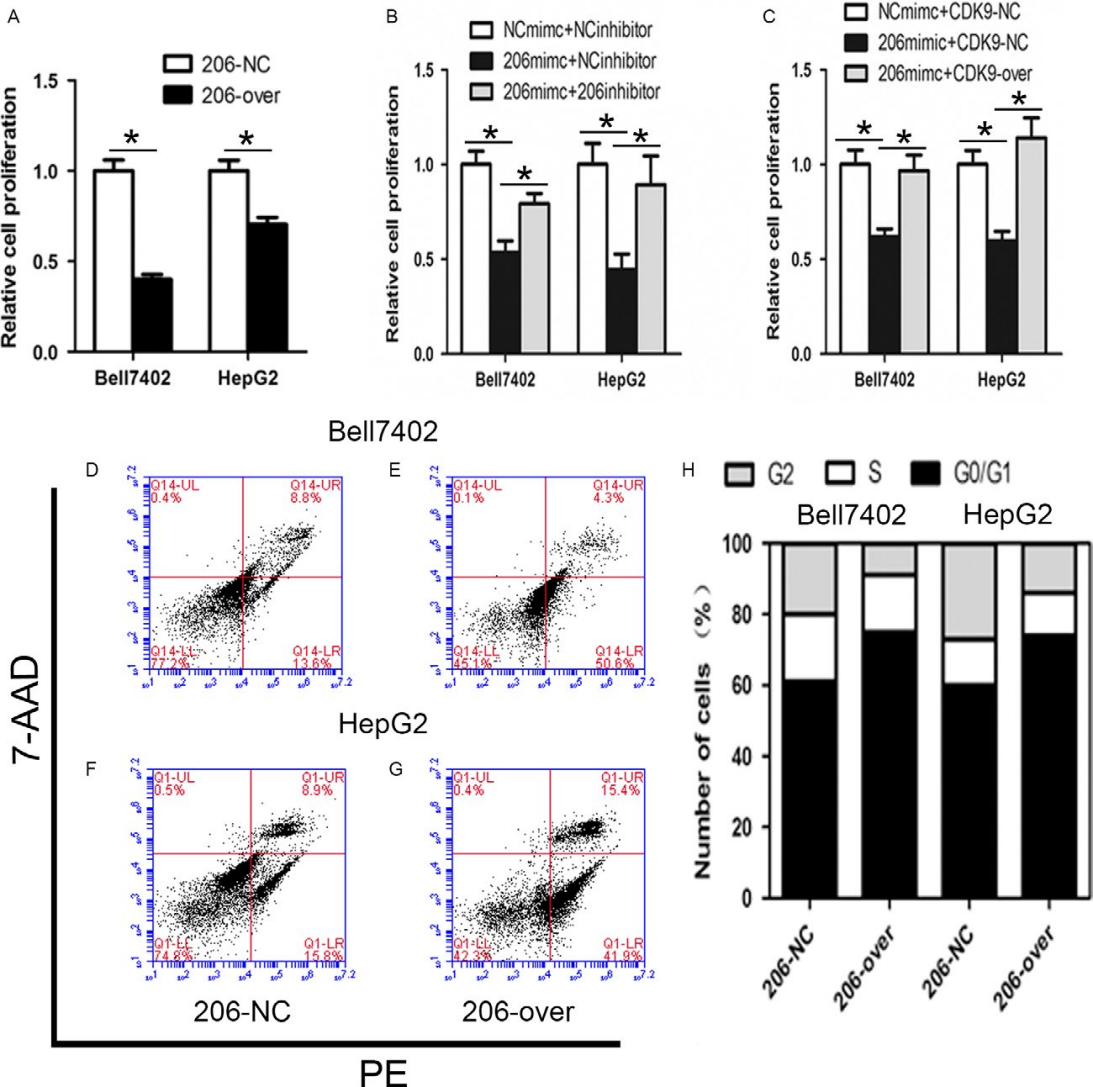
miR‐206 inhibits the proliferation, induces apoptosis, and cell cycle arrest in HCC cells. (A) Bell7402 and HepG2 cells were transfected withGV251‐miRNA‐206 and screened with G418 for 2–4 weeks to establish the stable cell lines.(B and C). Bell7402 and HepG2 cells were seeded in 96‐well plates after transfected with indicated plasmids and/or RNA oligos for 48 h. Then the CCK‐8 assays were performed for evaluating the cell proliferation. The apoptosis (D–G) and cell cycle phase distribution (H) were detected by flow cytometry. *P < 0.05.

Since miR‐206 targets CDK9, and CDK9 is a crucial factor for cell proliferation, we analyzed the effect of miR‐206 on the proliferation of HCC cells. The results showed that overexpression of miR‐206 in HCC cells significantly inhibited the cell proliferation (Bell7402, *P *=* *0.0017; HepG2, *P *=* *0.0122) (Fig. 5A). Furthermore, transient transfection of miR‐206 significantly suppressed HCC cell proliferation, which was dramatically attenuated by cotransfection of miR‐206 inhibitor (Bell7402, *P *=* *0.0143; HepG2, *P *=* *0.0322) (Fig. 5B) or GV230‐CDK9 (Bell7402, *P *=* *0.0236; HepG2, *P *=* *0.0367) (Fig. 5C). These results indicated that miR‐206 can inhibit HCC cells proliferation via targeting CDK9.

### miR‐206 induces apoptosis and cell cycle arrest in HCC cells

To address the mechanism by which miR‐206 represses the cell growth, flow cytometry was used to detect the effect of miR‐206 on cell apoptosis and cell cycle. As shown in Figure 5, overexpression of miR‐206 significantly induced apoptosis (Bell7402, *P *=* *0.0006; HepG2, *P *=* *0.0020) (Fig. 5D–G) and cell cycle arrest at G0/G1 phase (Fig. 5H), indicating that miR‐206 can suppress HCC cell growth via inducing apoptosis and cell cycle arrest.

### The shRNA and inhibitor of CDK9 have similar effects with that of miR‐206 in HCC cells

**Figure 6:**
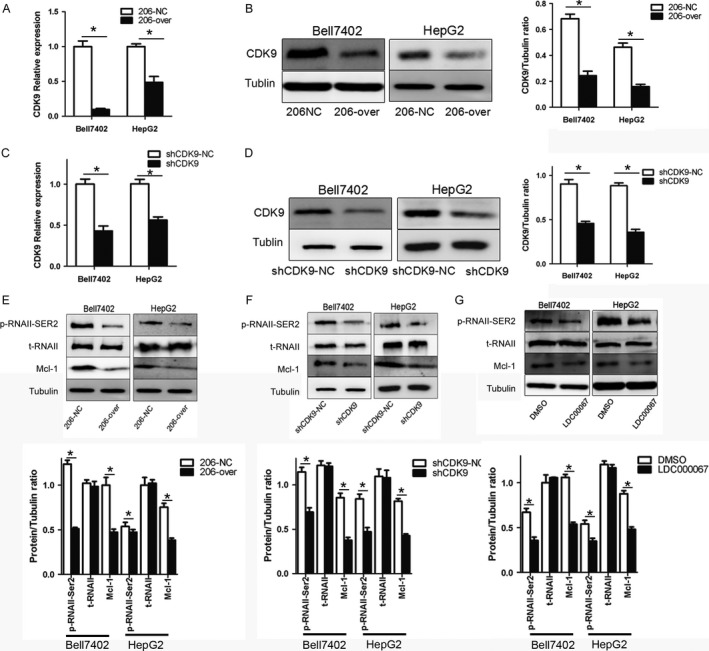
Overexpression of miR‐206, shCDK9, and LDC000067 suppresses the expression of CDK9 and its downstream genes. Bell7402 and HepG2 cells were transfected with GV251‐miRNA‐206 (A, E), sh‐CDK9 (C, D and F), and screened with G418 for 2–4 weeks to establish the stable cell lines or treated with 15 μmol/L LDC000067 (G) for 48 h. Then the levels of CDK9 mRNA were assayed by qPCR (A, C) taking β‐actin as a control, and the protein levels of CDK9, p‐RNAII, t‐RNAII, and MCL‐1 were detected by western blot (B, D–G) taking tubulin as the loading control. *P < 0.05.

**Figure 7:**
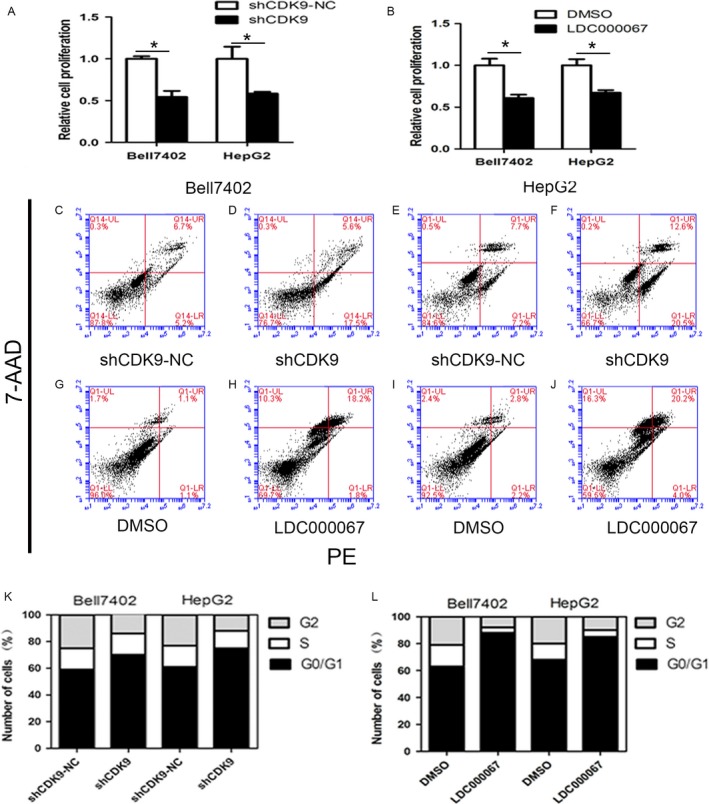
Inhibition of CDK9 by shCDK9 and LDC000067 represses the cell proliferation and induces apoptosis and cell cycle arrest in HCC cell lines. Bell7402 and HepG2 cells were transfected with sh‐CDK9 and screened with G418 for 2–4 weeks to establish the stable cell lines or treated with 15 μmol/L LDC000067 for 48 h, then the cell proliferation (A and B) was examined by CCK‐8 assays. The apoptosis (C–J) and the cell cycle phase distribution (K–L) were detected by flow cytometry. *P < 0.05.

To access whether the above miR‐206‐mediated effects were caused by knockdown of CDK9, shRNA (sh‐CDK9) and LDC000067 (CDK9‐inhibitor) were used to verify the effect. As shown in Figure 6A and B, overexpression of miR‐206 significantly suppressed the expression of CDK9 at mRNA (Bell7402, *P *=* *0.0417; HepG2, *P *=* *0.0086) and protein levels (Bell7402, *P *=* *0.0082; HepG2, *P *=* *0.0025) in HCC cells. Meanwhile, transfection of sh‐CDK9 (Fig. 6C and D) in HCC cells also markedly reduced the expression of CDK9 at mRNA (Bell7402, *P *=* *0.0235; HepG2 *P *=* *0.0037) and protein levels (Bell7402, *P *=* *0.0273; HepG2, *P *=* *0.0013). Moreover, the downstream targets of CDK9 including RNA polymerase II (Bell7402, *P *=* *0.0256; HepG2, *P *=* *0.0238) and Mcl‐1 (Bell7402, *P *=* *0.0233; HepG2, *P *=* *0.012) were dramatically repressed (Fig. 6E–G). Additionally, transfection of sh‐CDK9 and treatment with LDC000067 in HCC cells also markedly inhibited cell proliferation (Fig. 7A and B), induced apoptosis (Fig. 7C–J), and cell cycle arrest (Fig. 7K and L). The effects of sh‐CDK9 and CDK9 inhibitor were similar with that of miR‐206, indicating that the miR‐206‐induced proliferation repression, apoptosis, and cell cycle arrest in HCC cells were via downregulating CDK9.

## Discussion

Previous studies have revealed that CDK9 plays an important role in HCC development 31, 32, 33. However, it is still largely unknown about the regulatory mechanism of CDK9. In this study, we showed that miR‐206 significantly downregulates CDK9 in HCC cells, leading to cell proliferation inhibition, apoptosis, and cell cycle arrest.

miRNAs post‐transcriptionally regulate gene expression by promoting the degradation of target mRNAs or by inhibiting the translation of target mRNAs. Aberrant expression of miRNAs is involved in the development of human cancers 34. It has been reported that miR‐206 is dysregulated and linked to several types of cancers 35, 36, 37, 38. miR‐206 functions as a novel cell cycle regulator and tumor suppressor through regulation of various genes, such as the gap junction protein connexin 43 in breast cancer 39, 40, the apoptosis‐suppressing gene BCL‐2 in glioblastoma and HCC 22, 41, tRNA threonyl carbamoyl transferase subunit YRDC in bladder cancer 42, cell cycle activators cyclinD2 in laryngeal squamous 43 and gastric cancer 44, and the vascular endothelial growth factor VEGF in laryngeal cancer 45. In the present study, our results demonstrated that miR‐206 is markedly downregulated, while CDK9 is dramatically upregulated in HCC cells, suggesting that there is an inverse correlation between miR‐206 and CDK9. In a previous study, Xiao et al. show that miR‐206 inhibits ccRCC cell proliferation via directly targeting cell cycle‐related genes CDK4, CDK9, and CCND1 18. However, Georgantas et al. show that miR‐206 do not significantly affected CDK9 expression in melanoma 35, which suggested miR‐206 selectivity regulates CDK9 in distinct cancers. Thus, we address whether miR‐206 could serve as CDK9 suppressor in HCC. In this study, we defined the binding site of miR‐206 on the 3′ UTR of CDK9 mRNA and demonstrated that miR‐206 is a novel regulator of CDK9 in HCC cells. Thus, our results suggest that miR‐206‐CDK9 pathway might play a role in HCC.

CDK9, a member of the cdc2‐like serine/threonine kinase family, plays an important role in promoting cell proliferation and contribute to cancer 46, 47. CDK9 is a crucial transcriptional regulator, and might serve as a new and better therapeutic opportunity for cancers treatment 33, 48, 49. Consistent with previous studies, we found that inhibition of CDK9 could suppress HCC cells proliferation, induce cell cycle at G0/G1 arrest, and apoptosis, which could be rescued by overexpression of CDK9. Previous reports have shown that the proapoptotic effect of miR‐206 in HCC cells is, at least partially, dependent on Notch3‐mediated mitochondrial apoptotic signaling 50. Yuh‐Ying Yeh et al. show that the expression of Mcl‐1 is dependent on continuous RNAP II activity mediated by overexpression of CDK9 51. In this study, our results showed that miR‐206 could induce apoptosis by reducing the phosphorylation of RNAP II and the expression of Mcl‐1 via targeting CDK9. Moreover, the phosphorylation of RNAP II and the expression of Mcl‐1 were also significantly suppressed by shCDK9‐ or CDK9‐specific inhibitor. These results indicated that miR‐206 could induce apoptosis by directly inhibiting CDK9 ‐elated pathway in HCC cells.

In conclusion, miR‐206 is remarkably downregulated and is inversely correlated with CDK9 level in HCC cells. It is evident that miR‐206 functions as a tumor suppressor through blocking CDK9‐related pathway to induce apoptosis and inhibit HCC cell proliferation. The results may provide a novel regulation mechanism of miR‐206 in HCC, and suggest that targeting miR‐206‐CDK9 pathway may be a new approach for treatment of HCC.

## Conflicts of Interest

No potential conflicts of interest were disclosed.

## Supporting information


**Table S1.** The sequences for miRNA mimics and inhibitors.
**Table S2.** The sequences of primers used for PCR.
