# Supplementary material for: miR‐206 inhibits the growth of hepatocellular carcinoma cells via targeting CDK9
Source: Cancer Med. 2017 Sep 21;6(10):2398–409. doi: 10.1002/cam4.1188 (PMC5633544; doi:10.1002/cam4.1188)
Supplement: Supplementary file 1 — Table S1. The sequences for miRNA mimics and inhibitors. Table S2. The sequences of primers used for PCR. [file CAM4-6-2398-s001.docx]

Table S1The sequences for miRNA mimics and inhibitors.

| MiRNAs | Mimics | Inhibitors |
| --- | --- | --- |
| miR-206  NC | 5′-uggaauguaaggaagugugugg-3′  5′-uucuccgaacgugucacgutt-3′ | 5′-ccacacacuuccuuacauucca-3′  5′-caguacuuuuguguaguacaa-3′ |

Table S2 The sequences of primers used for PCR.

| Primer name | Sequence |
| --- | --- |
| WT 3′UTR forward  WT 3′UTR reverse  M 3′UTR forward  M 3′UTR reverse  hCDK9 forward  hCDK9 reverse  MCL-1 forward  MCL-1 reverse  hβ-actin forward  hβ-actin reverse  hu6 forward  hmiR-206 forward  hu6/hmiR-206 Reverse | 5′-acgcgtcgacttcaacttttaattctaacggac-3′ 5′-cgagctcctaaccctcagaaaacactgg-3′ 5′-gatcgccgtgtaattctagagtttttggcctaaccctcag-3′ 5′-ccggccgccccgactctagagtgtgtgcgcagaacaaaac-3′ 5′-cagcaaatcggacaattcct-3′ 5′-gccagcgaccaagtaaagag-3′  5′-cgacggcgtaacaaact-3′  5′-ggaagaactccacaaaccc-3′  5′-gtgaaggtgacagcagtcggtt-3′ 5′-gaagtggggtggttttagga-3′  5’-ctcgcttcggcagcac-3′  5’-tggaatgtaaggaagtgtgtgg-3′  5’-aacgcttcacgaatttgcgt-3′ |
